# No guarantees: planned oocyte cryopreservation, not quite an insurance policy

**DOI:** 10.1007/s00404-024-07654-4

**Published:** 2024-08-03

**Authors:** Sigal Klipstein, Lindsay Kelly, Sasmira Lalwani

**Affiliations:** 1 InVia Fertility, Chicago, IL USA; 2 Ferring Pharmaceuticals Inc., 100 Interpace Parkway, Parsippany, NJ 07054 USA

**Keywords:** Egg freezing, Fertility preservation, Ovarian stimulation, Family planning, In vitro fertilization

## Abstract

Planned oocyte cryopreservation (OC) has the potential to address the burden of the biological clock, giving women and individuals with ovaries more autonomy in choosing when to have children and with whom. In the United States, the annual number of OC cycles has grown significantly, yet many questions remain regarding planned OC. The field is starting to gather data on the clinical practice and social perspectives around planned oocyte cryopreservation, including the optimal age range at which to offer planned OC, what factors are most predictive of a successful outcome, and the optimal number of oocytes and ovarian stimulation cycles to achieve a live birth. There is a clear need for setting realistic expectations about the chance of success with OC; however, most patients have yet to return to thaw their oocytes, and outcomes data are limited. Clinical models have been developed to predict OC success based on surrogate markers such as age, number of oocytes retrieved, and anti-Müllerian hormone level. Patient education should emphasize the age-related decline in fertility, that eggs do not equal embryos, and that more than one cycle may be needed to obtain sufficient oocytes to have a reasonable chance of future success. While planned OC is not quite an insurance policy against future reproductive challenges, it provides the best option to date for expanding the reproductive window and maximizing reproductive options while navigating individual life circumstances in the context of family building.

## Introduction

While men often retain their reproductive capacity for the entirety of their lifetimes, women and individuals with ovaries generally lose the ability to reproduce, despite having decades of healthy life expectancy in front of them. This made evolutionary sense when life spans were significantly shorter, and women needed to survive to raise children to adulthood. With longer life spans, one can envision a modern biological imperative that justifies a broadening of the reproductive window.

Improvements in reproductive technologies have allowed for the cryopreservation of embryos initially and of oocytes more recently. The result has been an expansion of options for family planning, particularly for women who no longer need to be bound by ovarian senescence as a limitation to reproduction. While reproductive endocrinologists initially treated infertility with the single, immediate goal of achieving a live birth, today, patients may undergo assisted reproductive treatment (ART) with both immediate and long-term goals: achieving a live birth and/or cryopreserving eggs or embryos for future reproductive pursuits [1–3].

Over the past 40 years, the average age of women at the time of first birth in the US has increased by nearly 6 years, from 21.4 to 27.3 years of age [4, 5]. Furthermore, the number of women having their first child at or after age 35 has increased ninefold between 1972 and 2012 [6], and this trend continues. While the increasing age at first pregnancy is often attributed to a conscious choice to delay childbearing, many women report that their life circumstances (e.g., partnership, finances, and career) are not optimal for starting a family [1]. According to a 2013 US study, 88% of women noted the lack of a partner as one of the reasons that they delayed starting a family, with 24% citing professional considerations and 15% citing financial factors [7, 8]. These motivations continue to dominate women’s reproductive decision making [8]. A delay in childbearing may also be the result of a knowledge gap regarding the decline in fertility with increasing age, one that is seen even among female medical students [1, 9].

Planned oocyte cryopreservation (OC) has the potential to address the burden of the biological clock, giving women more control over their reproductive options. Indeed, trends in planned OC illuminate the importance of this option. In the US, the annual number of OC cycles has grown substantially over the past 10 years (+ 880% from 2010 to 2016) [10]. An increased uptake of planned OC was also reported during the COVID-19 pandemic [11–14]. SART data show an increase from 6090 OC cycles in 2014 to 24,560 OC cycles in 2021. Multiple factors may contribute to the increase in the utilization of OC, including post-pandemic changes in the approach to family and work, increased employer-based coverage, increasing availability of clinical outcomes data on OC, and a decrease in social stigma.

After ASRM lifted the “experimental” label from OC in 2012, at least for women facing gonadotoxic therapies, there was initial excitement about the procedure. It was presented as a medical solution to a social problem and was often misrepresented as being essentially a guarantee of future parenthood. However, there was very little data about the optimal age for OC, how many eggs and egg retrieval cycles would be needed to achieve a live birth, and the likelihood of the desired outcome. A decade later, women are now sharing what they wish they had known about planned OC, particularly that multiple cycles may be needed and that there is no guarantee of success. A survey from UCSF found that almost half of women felt decision regret around freezing eggs, particularly in cases when there was a low oocyte yield, they felt that their pretreatment counseling was inadequate, or they perceived a lack of access to emotional support during the process [15]. A more recent prospective survey from this same group showed that moderate to severe regret over the decision to freeze eggs was much lower, at 9%. Conversely, 51% of women who were initially counseled regarding the option of egg freezing but ultimately did not pursue treatment regretted this decision. For this subgroup, financial and time constraints were cited as the primary reasons for not availing themselves of this technology [16].

## Counseling and setting expectations

There is a clear need for setting realistic expectations about the chance of success with OC based on a woman’s age and the number of eggs that are produced. In addition, education needs to include the fact that eggs do not equal embryos, and that there is attrition during the process. Women should be counseled that more than one cycle is often recommended to obtain the number of oocytes needed to realize a reasonable chance of future success. Counseling should also address the increased risk of adverse outcomes associated with pregnancy at advanced maternal age, such as miscarriage, chromosomal abnormalities, fetal growth restriction, pre-term labor, pre-eclampsia, and gestational diabetes [17–21]. Proper counseling serves to empower women to make decisions that are best for them given their age and current situation. Informed consent should include an accurate assessment of expected outcomes, a clear delineation of risks, and the possibility that a pregnancy may not ultimately result, even despite favorable odds. It should also include a discussion of potential downstream costs, including those associated with oocyte thaw, fertilization, embryo culture, preimplantation genetic testing of embryos (PGT), and embryo transfer (ET). Providers are encouraged to disclose their own clinic-specific statistics for oocyte survival, fertilization, implantation, and live birth rate (LBR) in an effort to be transparent as patients choose whether and where to undergo their OC procedure.

Despite the increased number of planned OC cycles in recent years, most patients have yet to return to thaw their oocytes, and outcomes data are limited. The lack of data adds to the difficulty in appropriately counseling patients. Clinical models have been developed to predict OC success based on the relationship between age and the number of mature oocytes retrieved on live birth outcomes [22]. Though models may provide guidance, their utility is limited as they do not account for variability in vitrification and thaw technique as well as downstream IVF factors such as sperm quality, culture protocol, and uterine factors. Finally, there are some women who will never return to use their oocytes, and it is not clear if women who return are different than ones who do not, thus potentially skewing outcomes data. One study suggested that women with poor ovarian reserve, as delineated by the number of oocytes cryopreserved, are more likely to return to utilize them [23]. Another study looked at the impact of employee benefits for planned OC on its utilization, finding that employees (medical residents) were willing to make career decisions based on the potential for employer-sponsored planned OC [24].

## Planned OC outcomes data in the US: what have we learned?

Outcomes studies from planned OC remain limited. Cascante et al. summarized their experience with planned OC at the NYU Langone Center between 2005 and 2020 [25]. The study included 543 patients (median 38.3 years of age), 800 OC cycles, 605 oocyte thaws, and 436 transfers. A median of 12 oocytes were frozen per patient, and the median time between the first OC cycle and oocyte thaw was 4.2 years. LBR was 51% for patients whose first OC cycle was performed before 38 years of age, 34% for patients whose first OC cycle was performed between 38 and 40 years of age, and 23% for patients 41 years of age or older. No live births were reported for oocytes cryopreserved at or after 44 years of age. Reassuringly, the duration of cryopreservation did not influence LBR outcomes. A later publication by this group estimated that cryopreserving at least 20 oocytes at < 38 years of age yields a 70% chance of achieving at least one live birth [26].

Leung et al. 2021 [27] reported retrospective, observational data that included 921 patients (1265 cycles) with a mean age of 36.6 years who underwent planned OC from 2006 to 2020. 14.7% of patients’ oocytes were cryopreserved using a slow-freeze technique prior to the laboratory’s transition to vitrification in 2010. In their cohort, patients over the age of 38 years cryopreserved fewer oocytes than those younger than 38 years (15.2 vs. 18.4 oocytes). During the study period, 7.4% of patients returned to thaw their cryopreserved oocytes. Patients who cryopreserved oocytes after 38 years of age were more likely to use them and returned after a shorter time (mean of 3.2 years) than patients under 38 years of age (mean return time of 4.1 years). Of patients who returned to thaw, 32% achieved a live birth; 40% of patients who cryopreserved oocytes before 38 years of age achieved a live birth compared to 25% of patients who cryopreserved oocytes after 38 years. No patient over 40 years of age at the time of cryopreservation had a live birth. A more recent meta-analysis reported that 11.1% of women returned to use their cryopreserved oocytes, of which 28% achieved a live birth [28]. In this cohort, the live birth rate upon returning was 52% for women 35 years of age or younger vs. 19% for those 40 years of age or older [28].

## Predicting success of planned OC

The Brigham and Women’s Hospital Egg Freezing Counseling Tool was developed to help predict the chance of OC success based on age and number of mature oocytes retrieved [22]. One strength of the model is that it incorporates PGT-A and euploidy data; however, it relies on data extrapolated from oocyte donors and fresh IVF cycles in patients with normal ovarian reserve, which may overestimate the success of OC among older women with declining ovarian reserve. Oocyte thaw survival rates and euploidy assumed by the model are 95% for those under 36 years of age, which is much higher than that predicted by Cascante et al. at the NYU Langone Center [25, 26]. Multiple OC cycles may still be necessary to cryopreserve a sufficient number of oocytes to achieve a live birth. Multiple OC cycles may be necessary to achieve a live birth. Maslow et al. [29] developed a model based on retrospective data of planned OC cycles to estimate the number of oocytes needed to achieve a 50%, 60%, or 70% LBR. A cutoff for anti-Müllerian hormone (AMH) of > 1.995 ng/dL predicted a LBR of 60% with 1 cycle, regardless of age (*p* < 0.001), and women under 37.5 years of age were more likely to reach the 60% estimated LBR with 1 cycle, regardless of AMH (*p* < 0.001). Until predictive markers of oocyte developmental capacity are available, there will always be biologic variability that cannot be fully captured by models. How the gamete is handled during freezing and thawing, as well as the fertilization and culture procedures used, vary from clinic to clinic, and contribute to success rates. Further complicating success prediction is the fact that many women have yet to return to utilize their cryopreserved oocytes; as such, we expect that accurate models for predicting OC success rates will evolve over time [30].

## AMH as a predictor of success of planned OC

As a marker of ovarian reserve, AMH levels may be a useful tool for predicting egg yield for planned OC. AMH levels do not, however, predict the chance of unassisted conception at a given point in time, whether present or future. Linear surrogate markers for ovarian reserve that decline in predictable ways would be helpful in counseling women regarding the optimal timing of OC. Unfortunately, neither AMH nor any other currently available lab test can be utilized in this manner. The existing data on AMH and OC outcomes is not truly epidemiologic data, and there is no longitudinal data available on AMH levels throughout the reproductive lifespan. In addition to significant interassay variability, AMH fluctuates within individual patients. While on a population level AMH decreases over long periods of time with depletion of the ovarian reserve, it is unclear how to interpret a low or decreasing AMH on an individual patient basis in the short term. Furthermore, a high AMH level in the present does not predict any type of window during which egg yield will be high or remain high and cannot be used as a marker to determine if delaying planned OC is a reasonable option. One of the few longitudinal datasets available, the Nurses’ Health Study [31], found that women who had early menopause had lower AMH at age 35 than those who did not, but it is difficult to determine how to utilize this information to help women looking to understand either their current or future fertility.

At-home AMH testing has been growing in popularity, but results can have a dichotomous effect. Test results can increase stress regarding fertility or may end up encouraging women to delay childbearing. Women are hearing messages that “AMH is indicative of fertility” and that as long as the number is within the normal range, they do not need to worry. Unfortunately, at-home AMH test results are often provided with little context or counseling and are being inappropriately construed as more predictive than they actually are.

## What is the optimal age for planned OC?

In the US, the average age of women undergoing planned OC has decreased over time (36.7 years in 2010 vs. 34.7 years in 2016) [10]. Age is the strongest predictor of oocyte quality and OC outcomes, but may be modified by AMH. From a purely biological perspective, egg quality is better at a younger age. This has long been known given the field’s experience with autologous and egg donor cycles. It is for this reason that ASRM recommends that egg donors be under 35 years of age [32]. High-risk groups (e.g., *FMR1* carriers), women with endometriosis, and those with a history of exposure to potentially gonadotoxic therapies will likely benefit from pursuing OC at younger ages. However, planned OC is costly and includes storage fees and additional downstream costs (e.g., thaw, fertilization, PGT, ET). From a cost/benefit perspective, the ideal age to undergo OC will depend on balancing the higher success at a younger age against the decreased chance that younger women will ultimately return to use the eggs. Studies have demonstrated that between 7 and 16% of women return to utilize their cryopreserved eggs; however, utilization rates may change as more outcomes data become available [27, 33–35].

A 2015 study demonstrated that among women delaying childbearing until age 40 years, planned OC can reduce the cost per live birth [36]. Another recent study calculated that planned OC at 33 years of age with thaw at 43 years of age was more cost-effective than 3 IVF cycles with PGT-A at age 43 [37]. However, the psychological benefit that planned OC may provide women, regardless of whether they use their oocytes, should not be underestimated.

The increase in coverage for planned OC is also skewing conversations about cost and timing. Women may choose to pursue OC based on whether they have insurance coverage through their employer. Women may opt to expedite OC due to the fear of losing this benefit after a job change or alteration of benefits at their current employer.

## The impact of advanced maternal age on pregnancy risks and the wellbeing of offspring

Given that many women who will ultimately return to use their cryopreserved oocytes will do so at an advanced maternal age, the risks of reproduction with advancing age should be included in the initial counseling of women interested in OC [38]. These risks, which are particularly pronounced in women over the age of 45, include a higher incidence of cesarean deliveries, gestational diabetes, hypertensive disorders, pre-term delivery, placental abruption, and postpartum hemorrhage [39]. Children born to parents of advanced age are also more likely to experience early parental death, and this should be a part of the discussion when physicians initially counsel patients. Children experiencing parental death suffer from higher rates of depression, anxiety, post-traumatic stress disorder (PTSD), alcohol/drug problems, social withdrawal, and lowered self-esteem [40]. Taking into consideration the risks to both pregnancy and the health and wellbeing of offspring, fertility centers should determine an upper age limit at which oocytes may be thawed and utilized for pregnancy [38].

## Conclusions and open questions

Planned OC holds promise in expanding reproductive options for women who wish to prolong their fertile window. While planned OC cannot ensure a future pregnancy, it is currently the most effective option available to achieve this goal. Appropriate counseling based on the latest research is critical to obtain fully informed consent from women considering this option. As knowledge improves, counseling will better be able to identify which women stand to benefit the most from this technology. At present, many open questions remain. These include the optimal age range at which to offer planned OC, what factors are most predictive of a successful outcome, the optimal number of oocytes based on a woman’s age, and the number of ovarian stimulation cycles that should be undertaken.

Planned OC expands women’s reproductive autonomy and allows them to make decisions that are aligned with their personal values vis-à-vis family building. As a matter of reproductive justice, planned OC should be offered to all women who wish to build a family, and ways to provide this option broadly should be explored.

In any cost/benefit analysis, considerations such as the strength of a woman’s desire to create a family and the optimal number of children she would like to include in that family should be considered when weighing clinical decisions. Relationship status, professional aspirations, and individual health should also be part of the equation.

Planned OC may decrease the need for use of third-party reproduction including oocyte donation, and this cannot be excluded from such analyses. Finally, planned OC will overcome certain challenges inherent in embryo creation, namely, that it involves two gamete providers who may not be aligned in their reproductive goals. There may indeed be a time when OC is preferable to embryo creation for the purpose of maximizing reproductive options. Research will help determine the number of eggs that need to be thawed at any given time to get as close as possible to creating one embryo at a time and avoid the ethical and practical issues inherent in embryo cryopreservation. Ideally, effective models will be developed to guide the number of oocytes that should be thawed based on an individualized patient model that can predict euploid embryo yields.

There are several scenarios in which women would not utilize some or all of their previously cryopreserved oocytes. These include women who conceive spontaneously or with the help of other assisted reproductive technologies, women who complete their family plan without using all of their cryopreserved oocytes, and women who opt not to have children for personal reasons or when pregnancy is not medically advisable. In these cases, women may choose to donate their unused oocytes for reproduction or research.

Several additional questions include: is it unethical to offer planned OC if the likelihood of live birth is low? Is it better to counsel these patients to use donor eggs or consider adoption? Furthermore, is the definition of success for planned OC different than that for IVF (i.e., live birth)? For example, patients considering planned OC may be looking for peace of mind if they are unpartnered, they may be looking for a more cost-effective option than IVF with PGT-A at a more advanced age, or they may be planning for the future possibility of expanding their family. Answering the many remaining questions and providing evidence-based counseling for patients will require large, longitudinal data analyses to identify accurate predictors of OC outcomes. The field continues to explore ways to identify oocyte competency, which will have a significant impact on how patients pursuing planned OC are counseled and treated. It is critical that providers continue to set realistic expectations and educate patients about age-related declines in fertility and options for future family building.

While planned OC is not quite an insurance policy against future reproductive challenges, it provides the best option to date for expanding the reproductive window and allowing women to keep their reproductive options open as they navigate their individual life circumstances in the context of family building.

## References

[1] American Society for Reproductive Medicine (2018) Planned oocyte cryopreservation for women seeking to preserve future reproductive potential: an ethics committee opinion. Fertil Steril 110(6):1022–1028. 10.1016/j.fertnstert.2018.08.027 30396539 10.1016/j.fertnstert.2018.08.027

[2] Ethics Committee of the American Society for Reproductive Medicine (2024) Planned oocyte cryopreservation to preserve future reproductive potential: an Ethics Committee opinion. Fertil Steril 121(4):604–612. 10.1016/j.fertnstert.2023.12.030 38430080 10.1016/j.fertnstert.2023.12.030

[3] Practice Committee of the American Society for Reproductive Medicine (2021) Evidence-based outcomes after oocyte cryopreservation for donor oocyte in vitro fertilization and planned oocyte cryopreservation: a guideline. Fertil Steril 116(1):36–47. 10.1016/j.fertnstert.2021.02.024 34148587 10.1016/j.fertnstert.2021.02.024

[4] Centers of Disease Control and Prevention. American women are waiting to begin families: average age at first birth up more than 3 years from 1970 to 2000 2002. https://www.cdc.gov/nchs/pressroom/02news/ameriwomen.htm. Accessed 5 Dec 2023

[5] Osterman MJK, Hamilton BE, Martin JA, Driscoll AK, Valenzuela CP (2023) Births: final data for 2021. Natl Vital Stat Rep 72(1):1–53 36723449

[6] Martinez GM, Daniels K (2023) Fertility of men and women aged 15–49 in the United States: national survey of family growth, 2015–2019. Natl Health Stat Report 179:1–22 36692386

[7] Hodes-Wertz B, Druckenmiller S, Smith M, Noyes N (2013) What do reproductive-age women who undergo oocyte cryopreservation think about the process as a means to preserve fertility? Fertil Steril 100(5):1343–1349. 10.1016/j.fertnstert.2013.07.201 23953326 10.1016/j.fertnstert.2013.07.201

[8] Tsafrir A, Holzer H, Miron-Shatz T, Eldar-Geva T, Gal M, Ben-Ami I et al (2021) “Why have women not returned to use their frozen oocytes?”: a 5-year follow-up of women after planned oocyte cryopreservation. Reprod Biomed Online 43(6):1137–1145. 10.1016/j.rbmo.2021.08.026 34686418 10.1016/j.rbmo.2021.08.026

[9] Armstrong AG, Woods M, Keomany J, Grainger D, Shah S, Pavone M (2023) What do female medical students know about planned oocyte cryopreservation and what are their personal attitudes? J Assist Reprod Genet 40(6):1305–1311. 10.1007/s10815-023-02845-5 37347348 10.1007/s10815-023-02845-5 PMC10310635

[10] Johnston M, Richings NM, Leung A, Sakkas D, Catt S (2021) A major increase in oocyte cryopreservation cycles in the USA, Australia and New Zealand since 2010 is highlighted by younger women but a need for standardized data collection. Hum Reprod 36(3):624–635. 10.1093/humrep/deaa320 33367704 10.1093/humrep/deaa320

[11] Huttler A, Koelper N, Mainigi M, Gracia C, Senapati S (2022) Impact of the COVID-19 pandemic on the perception of planned oocyte cryopreservation in the United States. F S Rep 3(2):145–152. 10.1016/j.xfre.2022.04.008 35529036 10.1016/j.xfre.2022.04.008 PMC9065715

[12] Martini AE, Jahandideh S, Williams A, Devine K, Widra EA, Hill MJ et al (2021) Trends in elective egg freezing before and after the COVID-19 pandemic. Fertil Steril 116(3):e220. 10.1016/j.fertnstert.2021.07.596 10.1016/j.fertnstert.2021.07.596

[13] Michelle Weidenbaum E, Cascante SD, DeVore S, Hodes-Wertz B, Grifo JA, Blakemore JK (2021) Lockdown uptick: did the SARS-CoV-2 pandemic generate an increase in planned oocyte cyopreservation (POC)? Fertil Steril 116(3):e218–e219. 10.1016/j.fertnstert.2021.07.593 10.1016/j.fertnstert.2021.07.593

[14] Raghunandan A, Vyas N, Aluko A, Spandorfer SD, Rosenwaks Z (2022) Impact of the COVID-19 pandemic on social oocyte cryopreservation trends. Fertil Steril 118(4):e30. 10.1016/j.fertnstert.2022.08.104 10.1016/j.fertnstert.2022.08.104

[15] Greenwood EA, Pasch LA, Hastie J, Cedars MI, Huddleston HG (2018) To freeze or not to freeze: decision regret and satisfaction following elective oocyte cryopreservation. Fertil Steril 109(6):1097–104 e1. 10.1016/j.fertnstert.2018.02.127 29807657 10.1016/j.fertnstert.2018.02.127

[16] Jaswa EG, Pasch LA, McGough A, Wong R, Corley J, Cedars MI et al (2023) Decision regret among women considering planned oocyte cryopreservation: a prospective cohort study. J Assist Reprod Genet 40(6):1281–1290. 10.1007/s10815-023-02789-w 37058259 10.1007/s10815-023-02789-w PMC10310667

[17] Frederiksen LE, Ernst A, Brix N, Braskhoj Lauridsen LL, Roos L, Ramlau-Hansen CH et al (2018) Risk of adverse pregnancy outcomes at advanced maternal age. Obstet Gynecol 131(3):457–463. 10.1097/AOG.0000000000002504 29420406 10.1097/AOG.0000000000002504

[18] Hartwig TS, Sorensen S, Jorgensen FS (2016) The maternal age-related first trimester risks for trisomy 21, 18 and 13 based on Danish first trimester data from 2005 to 2014. Prenat Diagn 36(7):643–649. 10.1002/pd.4833 27135649 10.1002/pd.4833

[19] Magnus MC, Wilcox AJ, Morken NH, Weinberg CR, Haberg SE (2019) Role of maternal age and pregnancy history in risk of miscarriage: prospective register based study. BMJ 364:l869. 10.1136/bmj.l869 30894356 10.1136/bmj.l869 PMC6425455

[20] Reddy UM, Ko CW, Willinger M (2006) Maternal age and the risk of stillbirth throughout pregnancy in the United States. Am J Obstet Gynecol 195(3):764–770. 10.1016/j.ajog.2006.06.019 16949411 10.1016/j.ajog.2006.06.019

[21] Waldenstrom U, Cnattingius S, Vixner L, Norman M (2017) Advanced maternal age increases the risk of very preterm birth, irrespective of parity: a population-based register study. BJOG 124(8):1235–1244. 10.1111/1471-0528.14368 27770495 10.1111/1471-0528.14368

[22] Goldman RH, Racowsky C, Farland LV, Munne S, Ribustello L, Fox JH (2017) Predicting the likelihood of live birth for elective oocyte cryopreservation: a counseling tool for physicians and patients. Hum Reprod 32(4):853–859. 10.1093/humrep/dex008 28166330 10.1093/humrep/dex008

[23] Fouks Y, Sakkas D, Bortoletto PE, Penzias AS, Seidler EA, Vaughan DA (2024) Utilization of cryopreserved oocytes in patients with poor ovarian response after planned oocyte cryopreservation. JAMA Netw Open 7(1):e2349722. 10.1001/jamanetworkopen.2023.49722 38165675 10.1001/jamanetworkopen.2023.49722 PMC10762568

[24] Murphy HG, Compton SD, Moravek MB, Rosen MW (2024) Impact of employer-covered planned oocyte cryopreservation on decision-making for medical training. J Assist Reprod Genet 41(2):385–407. 10.1007/s10815-023-02990-x 38008880 10.1007/s10815-023-02990-x PMC10894800

[25] Cascante SD, Blakemore JK, DeVore S, Hodes-Wertz B, Fino ME, Berkeley AS et al (2022) Fifteen years of autologous oocyte thaw outcomes from a large university-based fertility center. Fertil Steril 118(1):158–166. 10.1016/j.fertnstert.2022.04.013 35597614 10.1016/j.fertnstert.2022.04.013

[26] Cascante SD, Berkeley AS, Licciardi F, McCaffrey C, Grifo JA (2023) Planned oocyte cryopreservation: the state of the ART. Reprod Biomed Online 47(6):103367. 10.1016/j.rbmo.2023.103367 37804606 10.1016/j.rbmo.2023.103367

[27] Leung AQ, Baker K, Vaughan D, Shah JS, Korkidakis A, Ryley DA et al (2021) Clinical outcomes and utilization from over a decade of planned oocyte cryopreservation. Reprod Biomed Online 43(4):671–679. 10.1016/j.rbmo.2021.06.024 34474973 10.1016/j.rbmo.2021.06.024

[28] Hirsch A, Hirsh Raccah B, Rotem R, Hyman JH, Ben-Ami I, Tsafrir A (2024) Planned oocyte cryopreservation: a systematic review and meta-regression analysis. Hum Reprod Update. 10.1093/humupd/dmae009 38654466 10.1093/humupd/dmae009

[29] Maslow BL, Guarnaccia MM, Ramirez L, Klein JU (2020) Likelihood of achieving a 50%, 60%, or 70% estimated live birth rate threshold with 1 or 2 cycles of planned oocyte cryopreservation. J Assist Reprod Genet 37(7):1637–1643. 10.1007/s10815-020-01791-w 32418136 10.1007/s10815-020-01791-w PMC7376799

[30] Alteri A, Pisaturo V, Nogueira D, D’Angelo A (2019) Elective egg freezing without medical indications. Acta Obstet Gynecol Scand 98(5):647–652. 10.1111/aogs.13573 30758059 10.1111/aogs.13573

[31] Bertone-Johnson ER, Manson JE, Purdue-Smithe AC, Steiner AZ, Eliassen AH, Hankinson SE et al (2018) Anti-Mullerian hormone levels and incidence of early natural menopause in a prospective study. Hum Reprod 33(6):1175–1182. 10.1093/humrep/dey077 29659835 10.1093/humrep/dey077 PMC6366539

[32] American Society for Reproductive Medicine (2021) Guidance regarding gamete and embryo donation. Fertil Steril 115(6):1395–1410. 10.1016/j.fertnstert.2021.01.045 33838871 10.1016/j.fertnstert.2021.01.045

[33] Cobo A, Garcia-Velasco J, Domingo J, Pellicer A, Remohi J (2018) Elective and onco-fertility preservation: factors related to IVF outcomes. Hum Reprod 33(12):2222–2231. 10.1093/humrep/dey321 30383235 10.1093/humrep/dey321

[34] Cobo A, Garcia-Velasco JA, Coello A, Domingo J, Pellicer A, Remohi J (2016) Oocyte vitrification as an efficient option for elective fertility preservation. Fertil Steril 105(3):755–64 e8. 10.1016/j.fertnstert.2015.11.027 26688429 10.1016/j.fertnstert.2015.11.027

[35] Kakkar P, Geary J, Stockburger T, Kaffel A, Kopeika J, El-Toukhy T (2023) Outcomes of social egg freezing: a cohort study and a comprehensive literature review. J Clin Med. 10.3390/jcm12134182 37445218 10.3390/jcm12134182 PMC10342811

[36] Devine K, Mumford SL, Goldman KN, Hodes-Wertz B, Druckenmiller S, Propst AM et al (2015) Baby budgeting: oocyte cryopreservation in women delaying reproduction can reduce cost per live birth. Fertil Steril 103(6):1446-53 e1–2. 10.1016/j.fertnstert.2015.02.029 25813281 10.1016/j.fertnstert.2015.02.029 PMC4457614

[37] Bakkensen JB, Goldman KN (2021) After the thaw: when patients return to use cryopreserved oocytes. Fertil Steril 115(6):1437–1438. 10.1016/j.fertnstert.2021.03.029 33863555 10.1016/j.fertnstert.2021.03.029

[38] Ethics Committee of the American Society for Reproductive Medicine (2016) Ethics Committee of the American Society for Reproductive M Oocyte or embryo donation to women of advanced reproductive age: an Ethics Committee opinion. Fertil Steril 106(5):3–7. 10.1016/j.fertnstert.2016.07.002 10.1016/j.fertnstert.2016.07.002 27450186

[39] Yogev Y, Melamed N, Bardin R, Tenenbaum-Gavish K, Ben-Shitrit G, Ben-Haroush A (2010) Pregnancy outcome at extremely advanced maternal age. Am J Obstet Gynecol 203(6):558 e1-567. 10.1016/j.ajog.2010.07.039 20965486 10.1016/j.ajog.2010.07.039

[40] Blank NM, Werner-Lin A (2011) Growing up with grief: revisiting the death of a parent over the life course. Omega (Westport) 63(3):271–290. 10.2190/OM.63.3.e 21928600 10.2190/OM.63.3.e

